# A comparative study of the enzymatic hydrolysis of batch organosolv-pretreated birch and spruce biomass

**DOI:** 10.1186/s13568-018-0643-y

**Published:** 2018-07-10

**Authors:** Vijayendran Raghavendran, Christos Nitsos, Leonidas Matsakas, Ulrika Rova, Paul Christakopoulos, Lisbeth Olsson

**Affiliations:** 1 Industrial Biotechnology Division, Department of Biology and Biological Engineering, Chalmers University of Technology, Kemivägen 10, 412 96 Gothenburg, Sweden; 2 Biochemical Process Engineering, Chemical Engineering Division, Department of Civil, Environmental and Natural Resources Engineering, Luleå University of Technology, 971 87 Luleå, Sweden; 3 Department of Molecular Biology and Biotechnology, The University of Sheffield, Firth Court, Western Bank, Sheffield, S10 2TN UK

**Keywords:** Cellic CTec2, Cellulose-rich biomass, Delignification, Inhibitor-free biomass, Saccharification yield

## Abstract

A shift towards a sustainable and green society is vital to reduce the negative effects of climate change associated with increased CO_2_ emissions. Lignocellulosic biomass is both renewable and abundant, but is recalcitrant to deconstruction. Among the methods of pretreatment available, organosolv (OS) delignifies cellulose efficiently, significantly improving its digestibility by enzymes. We have assessed the hydrolysability of the cellulose-rich solid fractions from OS-pretreated spruce and birch at 2% w/v loading (dry matter). Almost complete saccharification of birch was possible with 80 mg enzyme preparation/g_solids_ (12 FPU/g_solids_), while the saccharification yield for spruce was only 70%, even when applying 60 FPU/g_solids_. As the cellulose content is enriched by OS, the yield of glucose was higher than in their steam-exploded counterparts. The hydrolysate was a transparent liquid due to the absence of phenolics and was also free from inhibitors. OS pretreatment holds potential for use in a large-scale, closed-loop biorefinery producing fuels from the cellulose fraction and platform chemicals from the hemicellulose and lignin fractions respectively.

**Electronic supplementary material:**

The online version of this article (10.1186/s13568-018-0643-y) contains supplementary material, which is available to authorized users.

## Introduction

There is an urgent need to produce fuels and chemicals from renewable resources, and drastic actions are required to combat the emissions of greenhouse gases, as underlined in the report by the Intergovernmental Panel on Climate Change (Houghton 2009; IPCC 2014). The increase in population and the transition to an urban lifestyle, with its concomitant dependency on technology and increase in economic growth, will place/is already placing enormous pressure on the global demand for energy (Smil 2004) and food (The World Bank 2014). The chemical industry contributes about 5.2 trillion USD per annum to the global economy, and the transition to bio-based processes is imperative if we are to reduce our dependence on petrochemical feedstocks (Tan et al. 2016; Robertson et al. 2017; Lange 2017).

Biomass is an abundant renewable feedstock (Perlack and Stokes (Leads) 2011; Limayem and Ricke 2012; Kluts et al. 2017). However, it requires pretreatment (McCann and Carpita 2015) to release sugars that can be utilized by microorganisms to produce the products of interest. Several pretreatment methods, such as acid/alkaline hydrolysis, dilute ammonia, liquid hot water, sulfur dioxide, are available for biomass deconstruction (Mosier et al. 2005), but these processes produce compounds such as hydroxyl methyl furfural (HMF), furfural and acetic acid, which are inhibitory to the microorganisms used for fermentation (Piotrowski et al. 2014, 2015), or to the saccharification enzymes (Ximenes et al. 2011). Organosolv (OS) pretreatment—proposed as early as 1931 (Kleinert and v. Tayenthal 1931) for delignification (Johansson et al. 1987; Sannigrahi and Ragauskas 2013; Brosse et al. 2017), has gained much interest recently (Pan et al. 2005; Nguyen et al. 2015; Guragain et al. 2016; Katsimpouras et al. 2017a, b). OS pretreatment provides three distinct streams: a solids stream enriched in cellulose, thus offering better digestibility by cellulases; a hemicellulose stream containing xylose and xylans (that can be used by pentose-utilizing yeasts or chemically converted to platform chemicals such as HMF); and finally, a lignin stream offering valorization through chemical and thermal conversion, thereby providing added value to the process.

Nitsos and coworkers (2016) recently demonstrated the efficient dissolution of lignin combined with extensive hemicellulose removal for spruce and birch, using batch OS (with ethanol as the solvent and sulfuric acid as the catalyst). In the present study, we used these OS-pretreated, cellulose-rich solid fractions to assess their hydrolysability using a commercial enzyme solution. We also carried out enzyme dosage studies on the OS-isolated/pretreated cellulose fractions that performed best during hydrolysis, to compare the yields with their steam-pretreated counterparts.

## Materials and methods

### Pretreatment

**Fig. 1:**
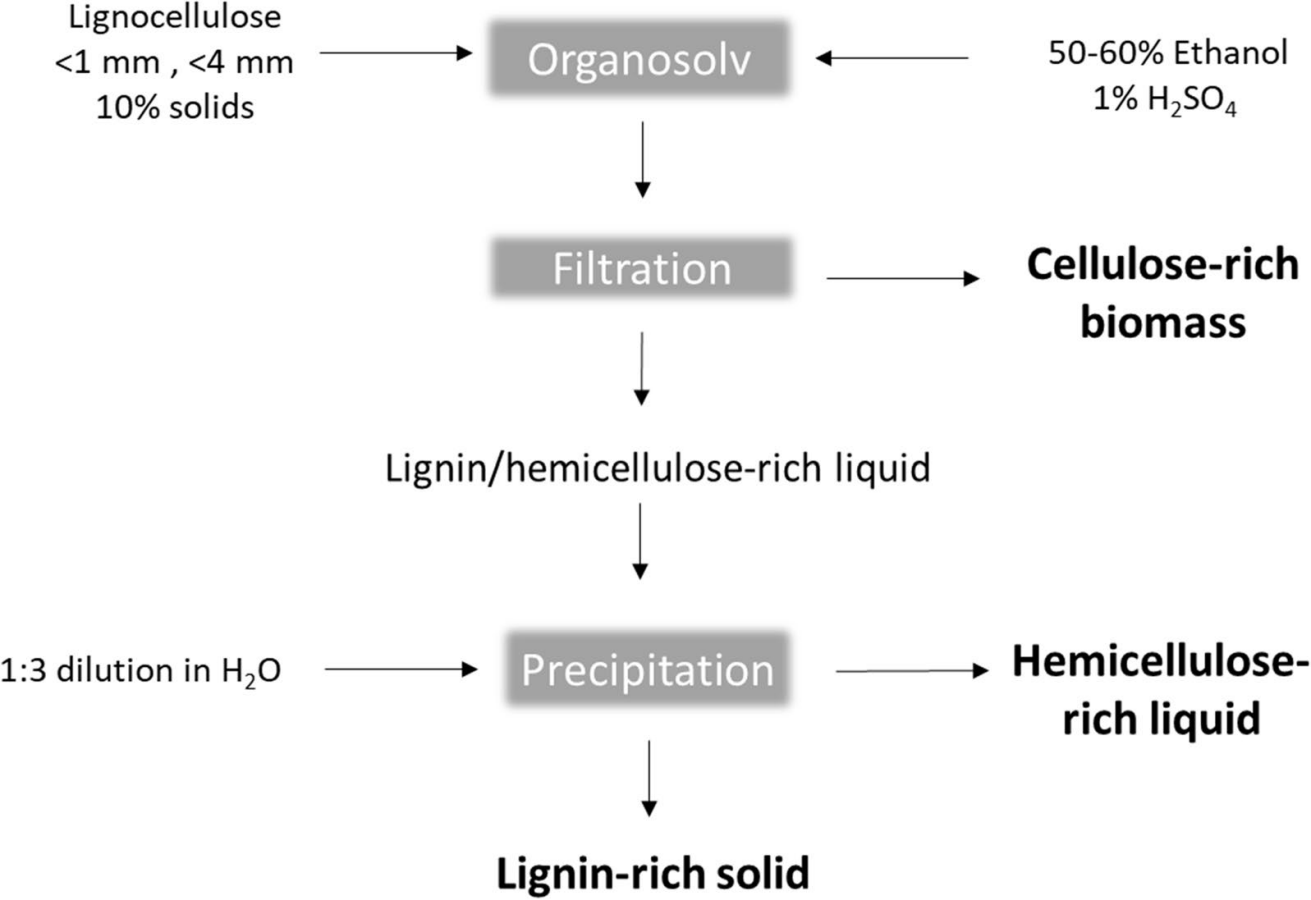
Schematic illustration of the pretreatment of birch and spruce using the organosolv method to obtain cellulose-rich biomass

Figure 1 shows a schematic of the batch OS pretreatment process used in our recently presented work on birch and spruce biomass fractionation (Nitsos et al. 2016). A total of 24 cellulose-enriched biomass samples were obtained by varying the concentration of the acid catalyst (0 or 1 wt% H_2_SO_4_), the duration of pretreatment (60 or 103 min), the ethanol concentration (50 or 60 wt%), and the particle size (< 1 or < 4 mm). Table 1 gives the conditions employed for biomass fractionation and the composition of the enriched biomass (Nitsos et al. 2016). Steam-exploded (SE) birch (200 °C, 5 min and 0.14 wt% H_2_SO_4_) and spruce (225 °C, 5 min and 0.5 wt% H_2_SO_4_) solids were used as controls.

**Table 1:** The conditions used in organosolv (OS) and steam explosion (SE) pretreatment together with the composition of the biomass obtained. From Nitsos et al. (2016)

	Biomass extracted with 50% (v/v) ethanol								Biomass extracted with 60% (v/v) ethanol			
Time	60 min, 182 °C				103 min, 182 °C				60 min, 182 °C			
Particle size	1 mm		4 mm		1 mm		4 mm		1 mm		4 mm	
H_2_SO_4_ (w/v)	0%	1%	0%	1%	0%	1%	0%	1%	0%	1%	0%	1%
Birch												
Cellulose	62.2	59.6	61.4	57.6	63.7	57.8	61.4	62.4	59.0	60.7	62.1	63.8
Hemicellulose	3.0	0.5	4.6	0.3	2.6	0.4	2.7	0.2	3.3	1.1	4.3	1.1
Lignin	16.0	24.1	14.6	21.8	17.5	25.9	15.8	23.7	16.8	15.7	13.8	16.0
Total (wt%)	81.2	84.1	80.6	79.8	83.8	84.2	79.9	86.3	79.0	77.5	80.2	80.9
Spruce												
Cellulose	52.5	56.1	53.8	58.0	54.5	55.9	56.1	55.2	50.3	69.1	49.1	67.3
Hemicellulose	5.0	2.0	6.4	2.4	5.6	3.5	7.3	2.8	13.2	1.2	14.5	2.8
Lignin	29.9	31.7	26.3	29.8	28.4	37.8	25.8	40.9	23.7	25.0	26.0	24.2
Total (wt%)	87.4	89.8	86.5	90.1	88.5	97.2	89.2	98.9	87.2	95.3	89.6	94.3

Composition of untreated birch (by wt%): 34.7% cellulose, 31.2% hemicellulose, and 18.7% lignin; composition of untreated spruce: 37.6% cellulose, 27.4% hemicellulose, and 32.6% lignin; composition of steam exploded birch: 57.2% cellulose, 12.1% hemicellulose, and 27.1% lignin (Matsakas et al. 2018); composition of steam exploded spruce: 38.2% cellulose, 53.1% lignin

### Enzymatic hydrolysis

Hydrolysis of the 24 samples with a dry matter content of 2% (w/v) was performed in cotton stoppered (and aluminum covered) 100 mL flasks, in a final volume of 40 mL. Citrate buffer at pH 4.8 and 50 mM final concentration was used to maintain the pH during hydrolysis. A stock solution of Cellic CTec2 (provided by Novozymes, Bagsværd, Denmark) was prepared, and appropriate volumes of the enzyme solution were used according to the dosages given in Table 1. A low enzyme loading of 20 mg enzyme preparation/g_solids_ (corresponding to 3 FPU/g_solids_) and an incubation time of 48 h was used during the initial screening experiments. Subsequently, dosages of 40, 80, 150, 300 and 400 mg enzyme preparation/g_solids_ were investigated. This preparation has a specific activity of 150 FPU/g (Wang et al. 2014), and thus 1 mg of enzyme preparation corresponds to 0.15 FPU. The flasks were incubated in a shaking water bath (OLS 200, Grant Instruments, Cambridge, UK), at 120 rpm (using an orbital arm of 9 mm radius) for 48 h at 50 °C. All experiments were performed in duplicate. The Analysis ToolPak in Microsoft Excel was used to determine the p-values using the student’s t test (two samples assuming equal variances, with a significance level of probability set at p < 0.05).

### Analytical determination

Samples obtained before and after hydrolysis (at 0 and 48 h) were filtered through 0.2 µm nylon syringe filters and stored at − 20 °C until further analysis. The glucose released was determined using HPLC at 80 °C with a Rezex column and a refractive index detector. The eluent was 5 mM H_2_SO_4_ and the flow rate 0.8 mL/min.

### Calculation of saccharification yield

The saccharification yield is defined as:

$$\eta = 100 *\left( {\frac{{{C_{glucose}}\;*{V_{liquid\;}}*\;0.90}}{{{m_{solids}}*\;{x_{cellulose}}\;}}} \right)$$

where *C*_*glucose*_ is the concentration of glucose obtained by HPLC, in g/L, *V*_*liquid*_ is the volume of the liquid used in hydrolysis; 0.90 is the correction factor for the addition of a molecule of water during the hydrolytic reaction; *x*_*cellulose*_ is the mass fraction of cellulose in the pretreated solids, and *m*_*solids*_ is the mass of pretreated solids in the experiment. All the masses used in the calculations were on a dry basis.

## Results

### Enzymatic hydrolysis of OS pretreated birch and spruce biomass

**Fig. 2:**
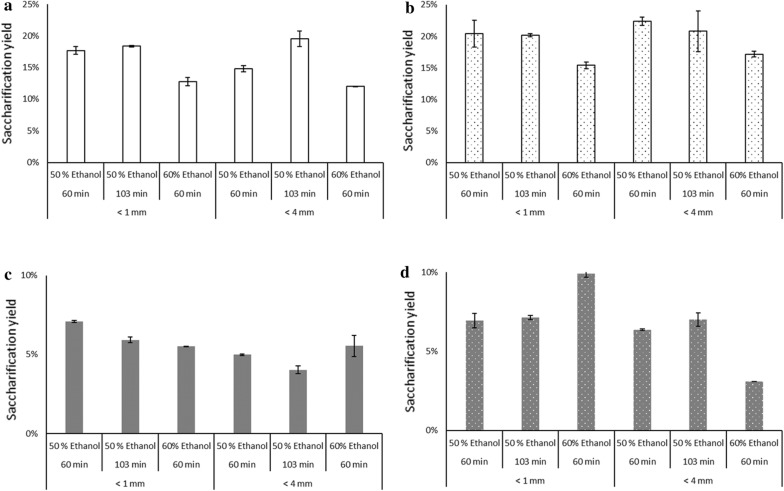
Saccharification yields of batch OS-pretreated birch and spruce biomass at 2% solids loading using 20 mg of the enzyme preparation/g_solids_; **a**, **b** Birch [without and with 1% H_2_SO_4_ catalyst pretreated with 50 or 60% ethanol at 60 or 103 min for a particle size of < 1 or < 4 mm;]. **c**, **d** Spruce [without and with 1% H_2_SO_4_ catalyst pretreated with 50 or 60% ethanol at 60 or 103 min for a particle size of < 1 or < 4 mm;]. See Table 1 for details of the pretreatment conditions. The results shown are the average of two experiments. Columns with dots indicate OS with acid catalyst

Nitsos and co-workers (2016) did an extensive characterization of batch OS treated birch and spruce biomass including the hemicellulose and lignin fraction. Here we screened the pretreated biomass from such a process for their hydrolysability aiming to study their potential for further use in microbial conversion processes. The saccharification yield of OS pretreated birch and spruce are shown in Fig. 2. No statistically significant differences were found between samples of the same wood type with and without the acid catalyst, or between various particle sizes, or ethanol concentration, although the use of the acid catalyst led to somewhat higher saccharification yields. However, differences between the wood types were statistically significant (p-value 0.00002 without acid, Fig. 2a and c; p-value 0.00032 with acid Fig. 2b and d). The maximum glucose concentration obtained after 48 h was 3.1 g/L (i.e. a yield of 0.14 g_glucose_/g_solids_) for birch and 1.6 g/L (0.076 g_glucose_/g_solids_) for spruce.

Additional file 1: Figure S1 shows the effects of each pretreatment variable on the saccharification yield. In general, increasing the concentration of ethanol in OS pretreatment reduced the saccharification yield for birch (p-value 0.0061), but not for spruce. The acid catalyst had a positive effect on the saccharification yield of both OS-pretreated birch and spruce. Particle size did not influence the saccharification yield for pretreated birch, but a reduction from < 4 to < 1 mm had a positive effect on pretreated spruce. The pretreatment time did not seem to affect the saccharification yields significantly. An average saccharification yield of 18% (20% with SE) was obtained with OS-pretreated birch, and 6% (5% with SE) with spruce.

### Enzyme dosage studies

**Fig. 3:**
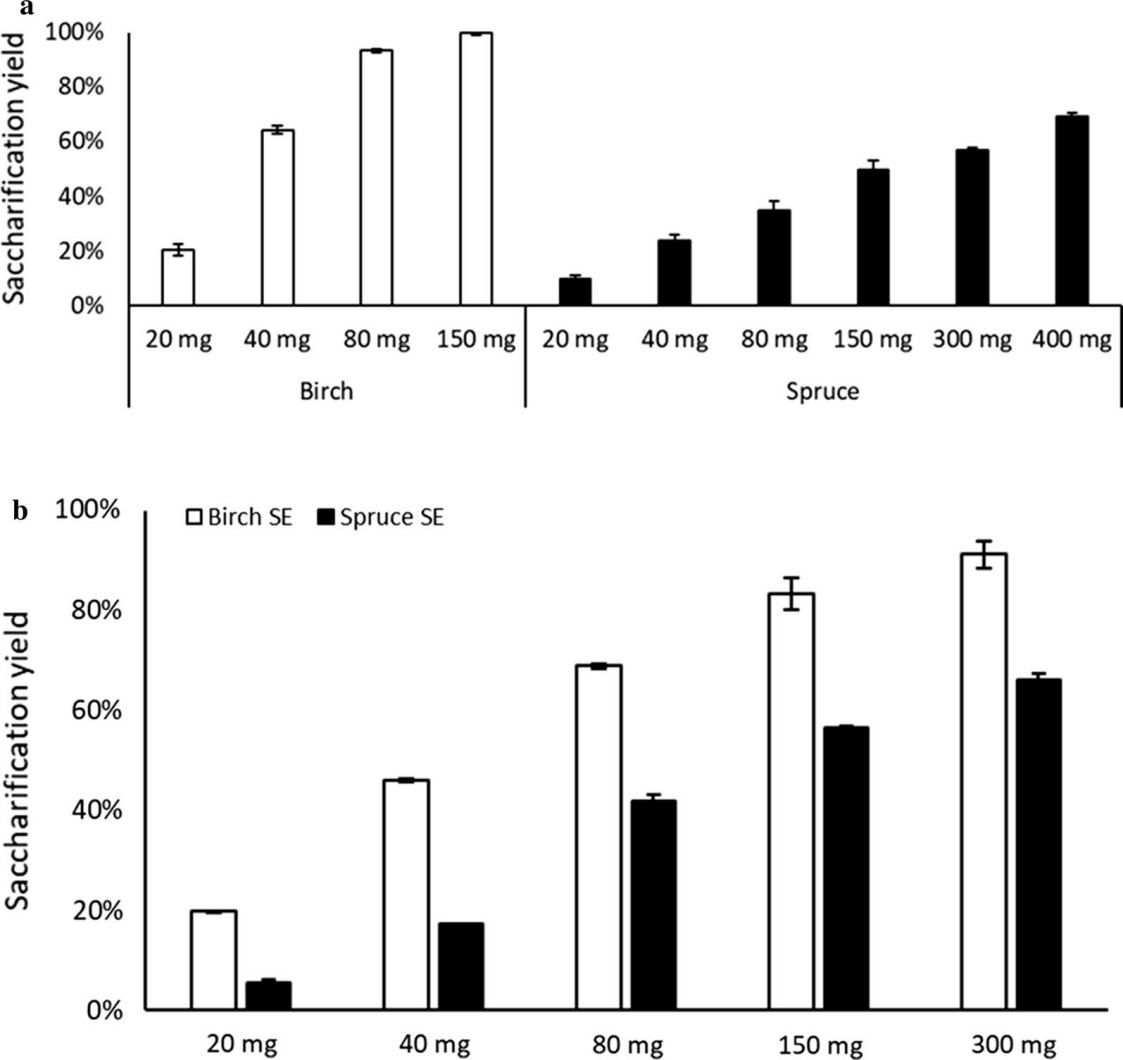
Results of the enzyme dosage studies on birch and spruce biomass at 2% w/v loadings pretreated with **a** OS and **b** SE [composition of SE birch samples and the saccharification yields taken from Matsakas et al. (2018)]

The effect of varying the enzyme dosage on the enzymatic saccharification of selected samples of OS-pretreated birch and spruce was also studied. The pretreatment conditions that gave the highest yield in the screening process were employed, i.e. 50% ethanol, 1% acid, for birch, and 60% ethanol and 1% acid for spruce, both at a particle size of < 1 mm. The results of these studies are shown in Fig. 3a. Doubling the enzyme dosage more than tripled the saccharification yield for pretreated birch, while pretreated spruce exhibited a modest 2.4-fold increase in yield. Almost 100% saccharification was achieved for birch when applying 150 mg enzyme preparation/g_solids_ whereas the highest saccharification yield obtained for spruce was 70%, when applying 400 mg enzyme preparation/g_solids_. The highest glucose concentrations achieved were 16 and 8 g/L for pretreated birch and spruce, respectively. The saccharification yields from the batch OS-pretreated samples were compared with those from samples pretreated with SE (Fig. 3b). Doubling the enzyme dosage lead to a 2.3-fold increase in saccharification yield for SE-pretreated birch and a 3.2-fold increase for SE-pretreated spruce. The maximum saccharification yields obtained were 91% for SE-pretreated birch and 66% for SE-pretreated spruce, when applying 300 mg enzyme preparation/g_solids_ (45 FPU/g_solids_).

## Discussion

As forest-based industries play a vital role in the Swedish economy, we focused on two woody raw materials: birch and spruce. Birch is a hardwood (angiosperm), widespread in the Northern Hemisphere. The lignin content varies between 18 and 25%, and consists of guaiacyl–syringyl units formed by the co-polymerization of coniferyl and sinapyl alcohols. Spruce, a softwood (gymnosperm), has a lignin content of more than 25%, and consists of guaiacyl units with a smaller proportion of unmethoxylated p-hydroxyphenyl units (Campbell and Sederoff 1996). It is well documented that OS pretreatment reduces the crystallinity of cellulose and enriches the cellulose fraction in the biomass through efficient delignification (Zhang et al. 2009; Zhao et al. 2009; Ju et al. 2014). High methane titers have been obtained by anaerobic digestion of OS-pretreated biomass (Matsakas et al. 2017). The superior quality of OS lignin, i.e. lower molecular weight and increased phenolic OH content (Nitsos et al. 2016), offers valorization through the production of biochemicals or as green phenol substitutes (Benar et al. 1999; Ruiz-Dueñas and Martínez 2009).

High saccharification yield of pretreated biomass is a prerequisite for subsequent bioconversion processes. Lignin present in the pretreated biomass is known to interfere with the enzymatic hydrolysis by forming a lignin-carbohydrate complex (Berlin et al. 2006). Nitsos and co-workers (2016) showed that their OS pretreatment resulted in a lignin removal efficiency of 69 and 62% for birch and spruce respectively. In the present work, we extended the work of Nitsos and co-workers to correlate the pretreatment variables with the saccharification yield. OS pretreatment enhanced the enzymatic digestibility of birch more efficiently than spruce; 95% saccharification was achieved with birch with 80 mg (12 FPU) enzyme preparation/g_solids_, while 70% was attained in spruce with 400 mg enzyme preparation/g_solids_ (60 FPU) (Fig. 3a). To compare, Obama and co-workers (Obama et al. 2012) used OS pretreatment of miscanthus, at a temperature of 170 °C, for 60 min using 80% (v/v) ethanol + 1% (w/w) H_2_SO_4_ and report a saccharification yield of ~ 55% at 40 IFPU of Celluclast^®^ 1.5 L/g of cellulose at a solids loading of 2% (w/v). It is likely that the high ethanol concentration they have used employed in the pretreatment prevented the complete hydrolysis of the biomass (compared to 95% for birch at 12 FPU and 56% for spruce at 45 FPU at 2% solids loading in our study). Smit and Huijgen (2017) used acetone–water (50% w/w) containing 40 mM H_2_SO_4_ at 140 °C and 120 min and report a saccharification yield of 78 and 16% for birch and spruce respectively at an enzyme loading of 10 FPU/g_solids_ (Accellerase TRIO) at 10% (w/v) solids loading (compared to a saccharification yield of 95 and 34% for birch and spruce at 12 FPU/g_solids_ at 2% solids loading in our study). As the saccharification yield is affected by the OS pretreatment process conditions, the type of solvent used in the OS, the type of enzyme cocktail used, as well as the consistency of solids during the hydrolysis process in small scale, it is harder to make a direct comparison with values reported in the literature.

As the hemicellulose fraction contains higher levels of glucuronoxylan (birch) or galactoglucomannan (spruce), the release of sugars of hemicellulosic origin was observed in birch and spruce respectively, during the saccharification process in samples not treated with the acid catalyst. This is an indication that the solubilization of hemicellulose was incomplete, and that treatment with an acid catalyst is needed for the efficient removal of hemicellulose. Thus, acid catalyst results in increased hemicellulose solubilization, decreased lignin—by cleaving the aryl-ether bonds (Sturgeon et al. 2014), resulting in increased cellulose content and saccharification yields. A decrease in saccharification yield observed with increasing amounts of ethanol indicates that there is an optimal ethanol concentration that offers the highest saccharification yield. Contrarily, low ethanol concentrations result in increased water activity, which creates more acidic conditions and promotes the cleavage of the α and β linkages in lignin (McDonough 1993). The difference in the saccharification yields seen in spruce and birch is due to differences in the lignin chemistry (different composition) and the non-productive interaction between the enzymes and lignin (Berlin et al. 2006). Few data are available on the effect of pretreatment time on the saccharification yield, but there is evidence that shorter pretreatment is favorable as the carbohydrate solubilization was not significantly increased when longer times were employed (Nitsos et al. 2016). In spruce, without the acid catalyst, increasing the duration of pretreatment decreases the saccharification yield, but the effect is reversed in the presence of the acid catalyst—increased saccharification yield with increased pretreatment time (Fig. 2c, d). Prolonged pretreatment might result in the formation of pseudo-lignin that could lead to an overestimation of lignin content and affect the accessibility of enzymes during enzymatic hydrolysis (Kumar et al. 2013). A retro-techno-economic analysis of the pretreatment, hydrolysis and fermentation process would be able to ascertain the process boundaries for economic feasibility (Longati et al. 2018) for further research and development.

In the final part of this study, OS pretreatment was compared with the traditional SE. Although the saccharification yield was at a comparable level for both SE and OS at the lower enzyme dosage (20 mg of enzyme preparation/g_solids_) for birch, significant differences could be observed at 80 mg of enzyme preparation/g_solids_ (69% for SE vs. 93% with OS) as seen in Fig. 3a and b. In the case of spruce, the differences were less prominent. However, due to the differences in cellulose content between OS- and SE-pretreated woody biomass, the yield of glucose was higher following OS than SE (0.74 vs. 0.53, and 0.37 vs. 0.24 g_glucose_/g_solids_, with 150 mg enzyme preparation/g_solids_ for birch and spruce, respectively). Thus, more glucose can be released per gram of OS-pretreated birch biomass upon hydrolysis. For example, with 20 wt% of OS-pretreated birch, the amount of glucose released would be ~ 150 g/L (compared to ~ 106 g/L with SE). Upon fermentation by yeast, this would give a theoretical ethanol yield of ~ 77 g/L (54 g/L with SE), demonstrating the potential for OS pretreatment over SE. Even though the cellulose conversion yields are similar between OS and SE, OS has the added advantage that the hemicellulose and lignin fraction can be collected as pure streams and could be utilized to produce other high-added value products, through chemical and biochemical routes.

In the present study, we used 2% w/v dry matter in the hydrolysis experiments, hence the theoretical amount of glucose that could be released is ~ 22 g/L. To achieve economically feasible titers of ethanol in a fermentation process (> 100 g/L), it is necessary to use high dry matter concentrations. As the aim of this study was to screen the pretreatment conditions, a low enzyme dosage and a low dry matter loading of 2% w/v were used as these are easier to handle and mix, leading to less experimental variation. Based on the maximum glucose concentration of 16 g/L (equivalent to 0.8 g_glucose_/g_solids_) observed after the hydrolysis of OS-pretreated birch, we suggest that similar hydrolytic yields could be attained at higher consistency using a free-fall mixer (Matsakas and Christakopoulos 2013; Katsimpouras et al. 2017b). However, spruce appears to be more recalcitrant, as the highest glucose concentration observed was 13 g/L (equivalent to 0.52 g_glucose_/g_solids_), and a saccharification yield of 70% at an enzyme dosage of 400 mg enzyme preparation/g_solids_.

One of the main drawbacks of the current batch OS process is the downtime between the runs. Thus, we are currently developing a continuous mode process that combines batch OS with explosive decompression of the biomass, which we hope will decrease the residence time and open the cellulose structure to improve enzyme access (Seidel et al. 2017). Life cycle assessment studies comparing the various pretreatment processes (Prasad et al. 2016) have shown that OS has no significant negative impact on the environment compared to SE, although the CO_2_ emission is higher due to the need to produce ethanol for the OS process (the ethanol can be distilled and recirculated, greatly reducing the net emission). Thus, OS appears to offer a promising method of pretreatment as the hydrolysate obtained is free from inhibitors, while simultaneously offering valorization of the hemicellulose and lignin stream. Retrofitting existing first-generation ethanol plants with the OS process to supply the sugar stream may help second-generation ethanol to become a commercial reality.

## Additional file

**Additional file 1: Figure S1.** Effects of the individual pretreatment variables on the saccharification yields. Ethanol concentration (50 or 60%), size of the wood chips employed (<1 mm or <4 mm), the presence or the absence of an acid catalyst (H_2_SO_4_) (0 or 1%), and the duration of pretreatment (60 min or 103 min). Open symbols: birch; filled symbols: spruce. **Table S1**. Hydrolytic yields reported in the literature using Cellic CTec2 for various pretreatment methods.

## Abbreviations

SE: steam explosion
OS: organosolv
