# Supplementary material for: A comparative study of the enzymatic hydrolysis of batch organosolv-pretreated birch and spruce biomass
Source: AMB Express. 2018 Jul 10;8:114. doi: 10.1186/s13568-018-0643-y (PMC6039347; doi:10.1186/s13568-018-0643-y)
Supplement: Supplementary file 1 — Additional file 1: Figure S1. Effects of the individual pretreatment variables on the saccharification yields. Ethanol concentration (50 or 60%), size of the wood chips employed (<1 mm or <4 mm), the presence or the absence of an acid catalyst (H2SO4) (0 or 1%), and the duration of pretreatment (60 min or 103 min). Open symbols: birch; filled symbols: spruce. Table S1. Hydrolytic yields reported in the literature using Cellic CTec2 for various pretreatment methods. [file 13568_2018_643_MOESM1_ESM.docx]

**AMB Express**

**A comparative study of the enzymatic hydrolysis of batch organosolv-pretreated birch and spruce biomass**

Vijayendran Raghavendran^1,3^; Christos Nitsos^2^; Leonidas Matsakas^2^; Ulrika Rova^2^_;_ Paul Christakopoulos^2^; Lisbeth Olsson^1^

^1^Chalmers University of Technology, Industrial Biotechnology Division, Department of Biology and Biological Engineering, Kemivägen 10, Gothenburg, SE-412 96, Sweden

^2^Biochemical Process Engineering, Chemical Engineering Division, Department of Civil, Environmental and Natural Resources Engineering, Luleå University of Technology, SE-971 87 Luleå, Sweden

^3^Deparment of Molecular Biology and Biotechnology, The University of Sheffield, Firth Court, Western Bank, Sheffield S10 2TN, UK

Corresponding author: [Lisbeth.olsson@chalmers.se](mailto:Lisbeth.olsson@chalmers.se); +46317723805

| 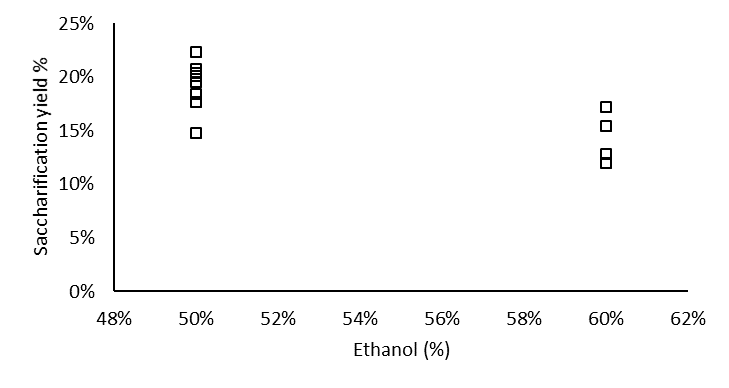 | 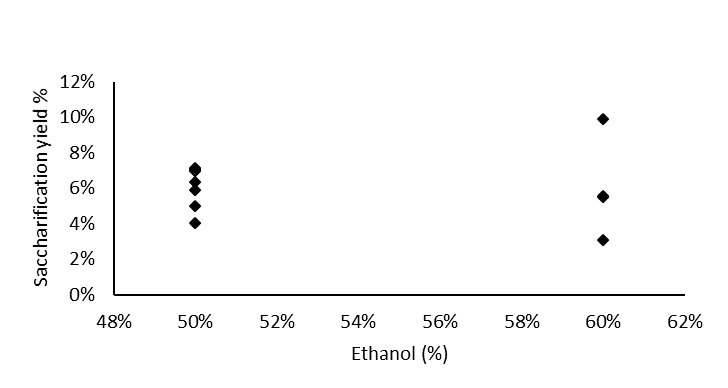 |
| --- | --- |
| 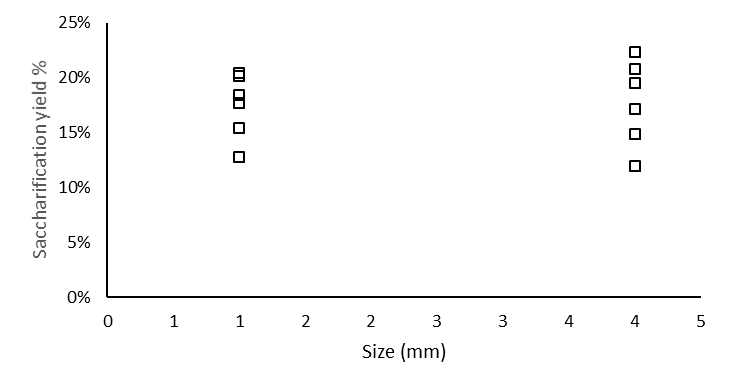 | 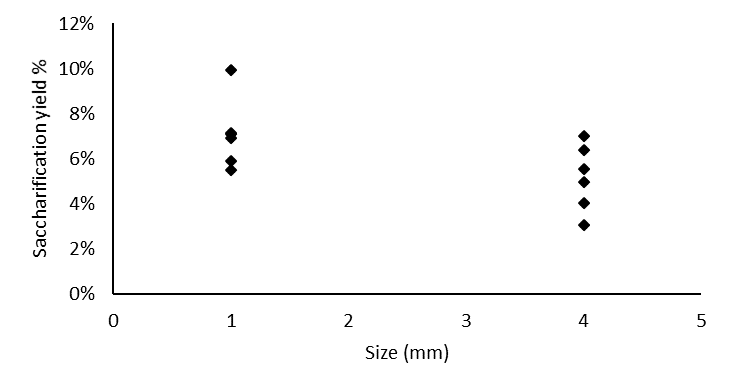 |
| 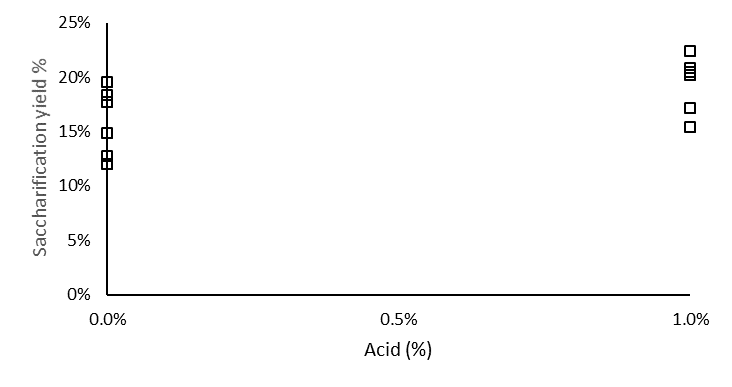 | 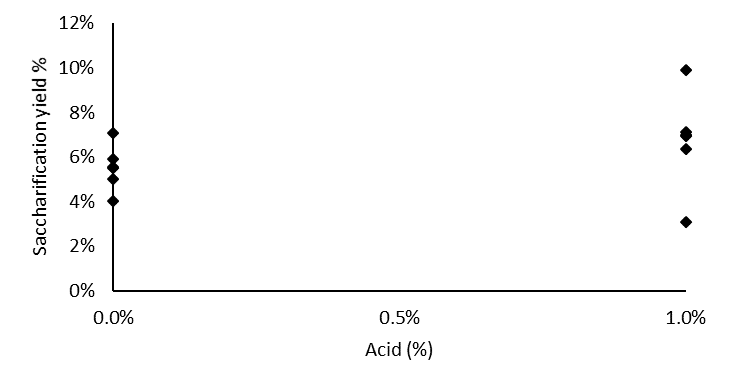 |
| 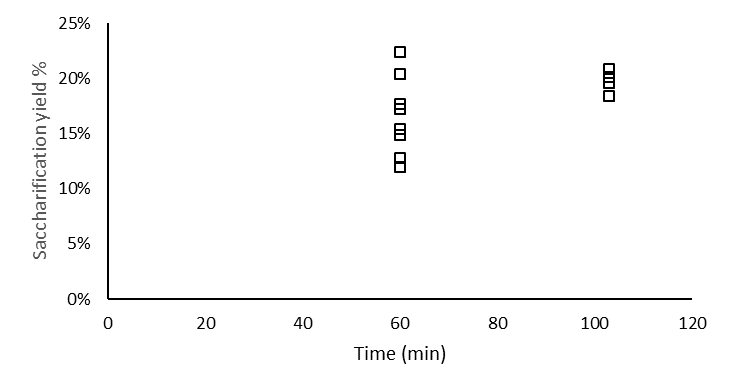 | 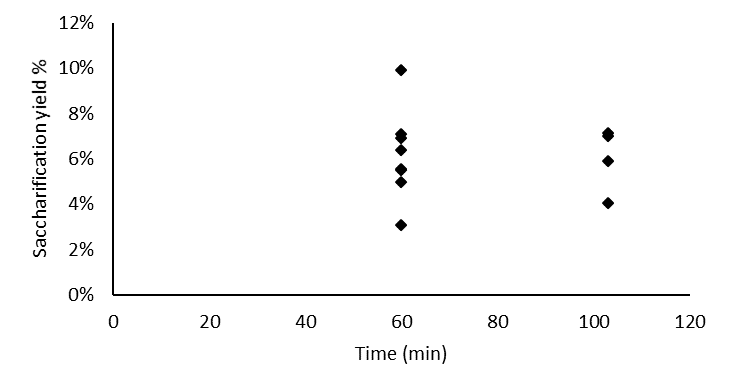 |

Figure S1: Effects of the individual pretreatment variables on the saccharification yields. Ethanol concentration (50 or 60%), size of the wood chips employed (<1 mm or <4 mm), the presence or the absence of an acid catalyst (H2SO4) (0 or 1%), and the duration of pretreatment (60 min or 103 min). Open symbols: birch; filled symbols: spruce.

Table S1: Hydrolytic yields reported in the literature using Cellic CTec2 for various pretreatment methods

| **Biomass** | **Solids loading** | **Yield** | **Cellic CTec2** | | |
| --- | --- | --- | --- | --- | --- |
| Avicel | 0.2%  0.2%  1% | 32.5%  50%  18% | 4 mg protein_/_g_solids_ (Ju et al. 2014)  20 mg protein_/_g_solids_ (Ju et al. 2014)  5 mg protein _/_g_cellulose_ (Resch et al. 2013) | |  |
| Beech wood (acetone-water OS) | 6% | 70% | 12 mg protein_/_g_solids_ (Katsimpouras et al. 2017) | |  |
| Birch (ethanol OS) | 2% | 64%  93% | 6 FPU_/_g_solids_ (This study)  12 FPU_/_g_solids_ (This study) | |  |
| Cocksfoot grass (wet acid explosion) | 10% | 88% | 20 mg protein/g_solids_ (Njoku et al. 2013) |  |  |
| Spruce (ethanol OS) | 2% | 24%  35% | 6 FPU/g_solids_ (This study)  12 FPU/g_solids_ (This study) | |  |
| Sugarcane bagasse (glycerol OS) | 2% | 82%  68% | 10 FPU/g_solids_ (Sun et al. 2016)  6 FPU/g_solids_ (Sun et al. 2016) | |  |
| Switch grass (dilute acid pretreated) | 2% | 75% | 20 mg protein/g_cellulose_ (Resch et al. 2013) | |  |

**References**

Ju X, Bowden M, Engelhard M, Zhang X (2014) Investigating commercial cellulase performances toward specific biomass recalcitrance factors using reference substrates. Appl Microbiol Biotechnol 98:4409–4420. doi: 10.1007/s00253-013-5450-4

Resch MG, Donohoe BS, Baker JO, Decker SR, Bayer EA, Beckham GT, Himmel ME (2013) Fungal cellulases and complexed cellulosomal enzymes exhibit synergistic mechanisms in cellulose deconstruction. Energy Environ Sci 6:1858. doi: 10.1039/c3ee00019b

Katsimpouras C, Kalogiannis KG, Kalogianni A, Lappas AA, Topakas E (2017) Production of high concentrated cellulosic ethanol by acetone/water oxidized pretreated beech wood. Biotechnol Biofuels 10:54. doi: 10.1186/s13068-017-0737-9

Njoku SI, Uellendahl H, Ahring BK (2013) Comparing oxidative and dilute acid wet explosion pretreatment of cocksfoot grass at high dry matter concentration for cellulosic ethanol production. Energy Sci Eng 1:89–98. doi: 10.1002/ese3.11

Sun FF, Zhao X, Hong J, Tang Y, Wang L, Sun H, Li X, Hu J (2016) Industrially relevant hydrolyzability and fermentability of sugarcane bagasse improved effectively by glycerol organosolv pretreatment. Biotechnol Biofuels 9:59. doi: 10.1186/s13068-016-0472-7
